# Impact of Perioperative Absolute Neutrophil Count on Central Line-Associated Bloodstream Infection in Children With Acute Lymphoblastic and Myeloid Leukemia

**DOI:** 10.3389/fonc.2021.770698

**Published:** 2021-11-23

**Authors:** Illya Martynov, Joachim Schoenberger

**Affiliations:** ^1^ Department of Pediatric Surgery, University Hospital of Freiburg, Freiburg, Germany; ^2^ Department of Pediatric Surgery, University of Leipzig, Leipzig, Germany; ^3^ Department of Pediatric Surgery, Helios Kliniken Schwerin, Schwerin, Germany

**Keywords:** tunneled central venous catheters, neutropenia, absolute neutrophil count (ANC), CLABSI, ALL—acute lymphoblastic leukemia, AML—acute myeloid leukemia, children

## Abstract

**Background:**

There is lack of evidence concerning safety of placement of tunneled central venous catheters (TCVCs) in neutropenic children with acute leukemias. Here, we evaluate the impact of absolute neutrophil count (ANC) at the time of TCVC placement on development of central line-associated bloodstream infections (CLABSI) in children with lymphoblastic (ALL) or myeloid leukemia (AML).

**Materials and Methods:**

A retrospective observational study of children undergoing TCVC placement at a tertiary referral hospital between January 2000 and December 2019 was performed. Traditional and competing-risks regression models were used to estimate the effect of perioperative ANC on development of CLABSI.

**Results:**

A total of 350 children (median age 6.4 [IQR: 3.1–10.9] years) underwent 498 consecutive TCVC implantations in neutropenic (*n* = 172, 34.5%) and non-neutropenic conditions (*n* = 326, 65.5%). The median length of observation per TCVC was 217.1 (IQR: 116.1–260.5) days with a total of 99,681 catheter days (CD). There were no differences in early (within first 30 days after TCVC placement) and overall CLABSI rates between neutropenic and non-neutropenic patients (HR 1.250, *p* = 0.502; HR 1.633, *p* = 0.143). We identified female sex (HR 2.640, *p* = 0.006) and the use of TCVC for treatment of relapsed leukemia (HR 4.347, *p* < 0.0001) as risk factors for early CLABSI and the use of double-lumen catheters (HR 2.607, *p* = 0.003) and use of TCVCs during leukemia relapse (HR 2.004, *p* = 0.005) for overall study period.

**Conclusion:**

The placement of TCVC in children with neutropenia undergoing anticancer therapy for acute leukemia is safe and not associated with an elevated rate of CLABSI.

## Introduction

Acute lymphoblastic leukemia (ALL) and acute myeloid leukemia (AML) are the two most common types of acute childhood leukemias accounting for approximately 30% of all pediatric malignancies (1). The intensive treatment of both ALL and AML requires long-term central venous access, e.g., a tunneled central venous catheter (TCVC). This facilitates the administration of chemotherapeutics, antibiotics, fluids, blood products, and parenteral nutrients. However, TCVCs are often associated with infective events, including local catheter exit-site infections (CESI) and central line-associated bloodstream infections (CLABSI). Both may delay or interrupt anticancer therapy, resulting in substantial morbidity and mortality (2–4). Furthermore, CLABSI were found to be the most costly healthcare-associated infections (5). As pediatric patients with hematooncological diseases often present with neutropenia (absolute neutrophil count [ANC] <0.5 × 10^9^/L or <1.0 × 10^9^/L) and are therefore *a priori* at a higher risk of infection, some studies have attempted to identify the effect of neutrophil levels at the time of catheter insertion on development of CESI and CLABSI. Several studies in pediatric patients with cancer have found an association between neutropenia at the time of catheter insertion and increased incidence of CESI/CLABSI, proposing low perioperative ANC as a potential risk factor for both (6–8). Consequently, some centers recommend TCVC placement only with ANC higher than 0.5 × 10^9^/L or even 1.0 × 10^9^/L. However, the literature is inconsistent regarding the association of perioperative neutropenia and increased incidence of CESI/CLABSI, with other studies showing no significant differences of CESI/CLABSI rates in neutropenic compared to non-neutropenic children at the time of catheter placement, indicating the safety of TCVC implantation also in the setting of neutropenia (9–11). Finally, most of the previous studies investigate the association between perioperative ANC and TCVC-associated infective complications in heterogeneous patient populations, which differ at baseline risk for CLABSI (12). Therefore, we aimed to investigate the impact of perioperative neutropenia on development of CESI and CLABSI in a large and well-defined cohort of highly vulnerable pediatric ALL and AML patients undergoing placement of TCVC.

## Methods

### Patients and Data Collection

In this retrospective, single-center cohort study, all consecutive pediatric patients (<18 years of age) with ALL or AML who underwent placement of indwelling tunneled central venous catheters (Groshong^®^ [closed-ended catheter with a valve], Hickman/Broviac^®^ [open-ended catheter] or port catheters) at the University Medical Center of Freiburg between January 1, 2000 and December 1, 2019 were included. Both catheter-naïve patients and patients with a prior history of catheter insertions were included. We used the Patient Data Management System (PDMS) to extract the data, including patient and device characteristics (patient´s age at TCVC placement, sex, underlying malignancy, date of TCVC implantation, revision, and explantation), laboratory (absolute neutrophil count), and microbiological tests.

### Preoperative Evaluation, Device Characteristics, and Placement Technique

Preoperative workup generally consisted of laboratory studies including complete blood count and coagulation tests. We did not consider ANC less than 0.5 × 10^9^/L as a contraindication for TCVC placement. A single-shot prophylactic perioperative antibiotic (e.g., cefuroxime) was administered to all patients irrespective of their ANC status. The TCVC placement took place in the operation theater under general anesthesia. All procedures were performed or supervised by surgical attendings. We did not use any in-line filters or anti-infective lock prophylaxis at the time of TCVC insertion. We used anatomical landmarks and the adapted Seldinger technique for catheter cannulation (13). All catheters were tunneled subcutaneously. The tissue ingrowth cuff was positioned at the distal end of the subcutaneous tunnel. In almost all cases, we used the right internal jugular vein for cannulation. The size of the catheter was determined by the age and weight of the patient. Correct positioning of the distal catheter tip between the superior vena cava and right atrium junction was verified by fluoroscopy. All operative procedures and applied techniques remained constant during the overall study period. Postoperative TCVC maintenance procedures were performed according to the standard operating procedure manual of our institution.

### Definitions

A CLABSI was defined according to the Centers for Disease Control and Prevention (LCBI 1 criteria, [laboratory confirmed bloodstream infection]) and the National Healthcare Safety Network criteria as a laboratory-confirmed bacteremia or fungemia originating from one or both lumens of the implanted device developing at least 48 h after TCVC placement and not related to an infection at another site (14, 15). Therefore, children who developed CLABSI within 48 h after TCVC placement were not considered as event of interest for the primary endpoint of the study. A recurrent CLABSI was defined as a catheter-associated infection after a time frame of 14 days following prior CLABSI. Consequently, positive blood cultures originating from catheter lumens occurring within the first 2 weeks after previous CLABSI were not considered as new CLABSI episodes. Neutropenia was defined as ANC < 0.5 × 10^9^ cells/L.

### Endpoints

The primary endpoint of our study was the effect of perioperativeANC (ANC < 0.5 × 10^9^ cells/L *vs.* ANC > 0.5 × 10^9^ cells/L) on development of (first) CLABSI (censored at premature catheter removal, death or last follow-up) within the first 30 days after TCVC placement. Analysis for differences in the primary endpoint was performed using the univariable (unadjusted) and multivariable (adjusted for the following co-variables: patient´s age at time of TCVC implantation, underlying malignancy, sex, purpose of catheter use [primary therapy or therapy for relapsed leukemia], type of TCVC, number of lumens, and size of catheter tube) survival models. Secondary endpoints included CLABSI incidence during overall study period, CESI incidence within 30 days period after TCVC implantation, and description of microorganisms detected during first CLABSI stratified by children’s neutropenic status peri-operatively and at the onset of the infection.

### Statistics

Categorical variables (e.g., sex and catheter type) were described using frequencies and percentages. Continuous variables (e.g., age and duration of catheter use) were reported as median and corresponding interquartile range (IQR). Fisher’s exact test was used for analysis of categorical variables and Mann–Whitney *U* test was used for continuous variables. The total number of catheter days (CD) per case was calculated as the total number of days from TCVC insertion to last follow-up (e.g., elective catheter removal, premature catheter explantation, and death). The complication rate (CR) per 1,000 CD was calculated as 1,000 times the number of complications divided by the total number of CD (16, 17). The risk of developing CLABSI during short-term (within first 30 days after TCVC placement) and overall follow-up period was estimated using the traditional Kaplan–Meier method (univariable/unadjusted analysis, crude HR) and Cox regression survival analysis (multivariable/adjusted analysis). Due to the presence of competing risks (catheter removal [elective *vs.* non-elective] and death), the risk of developing CLABSI during the short-term period and during the overall follow-up period was additionally estimated using competing-risks regression model (Fine & Gray [cumulative incidence function [CIF]) (18). The endpoint of interest in survival analysis was the time to first CLABSI. All consecutive CLABSI episodes were ignored in the analysis. All statistical tests were two-tailed and the tests were considered significant with *p* < 0.05.

### Ethics

The study protocol was approved by the Institutional Review Board of the University Hospital of Freiburg on December 8, 2020 (IRB, protocol number 20-1257). Written or verbal informed consent was not required.

## Results

### Study Population

Overall, 350 individual patients with a median age at time of catheter implantation of 6.4 [IQR 3.1–10.9] years (male 198 [56.6%] and female 152 [43.4%]), with ALL (*n* = 284, 81.1%) and AML (*n* = 66, 18.9%), were treated in our department during the study period. In these patients, 498 tunneled central venous catheters were placed, including Groshong (*n* = 340, 68.3%), Hickman-Broviac (*n* = 129, 25.9%), and port (*n* = 29, 5.8%) catheters. Among these devices, 399 (80.1%) were used for primary therapy, 75 (15.1%) for first, 12 (2.4%) for second, and one (0.2%) for third relapse of disease. Eleven TCVCs (2.2%) were used for both primary and relapsed leukemia treatments. The median CD per TCVC case was 217.1 days (IQR: 116.4–260.5) with a total of 99,681 CD for all TCVCs. Perioperative neutropenia was observed in 34.5% (*n* = 172) of cases. The neutropenic at TCVC insertion patients were significantly younger (median age 5.2 [IQR: 2.7–8.1] years, *p* < 0.0001) than non-neutropenic children and had smaller (66.3% of ≤7 French catheters, *p* = 0.0006) single-lumen (61.6%, *p* = 0.0266) devices implanted. Moreover, patients with perioperative neutropenia had more frequently primary disease (90.7%, *p* < 0.0001) and had a higher proportion of Hickman-Broviac catheters (34.9%, *p* = 0.0038). Detailed characteristics of children with and without neutropenia at the time of TCVC insertion are summarized in **Table 1**.

**Table 1:** Clinical and device characteristics of study population.

Characteristics	Total cohort *n* = 498	Neutropenic at TCVC implantation		
		Yes *n* = 172 (34.5%)	No *n* = 326 (65.5%)	*p*-value
**Age**, years, Median (IQR)	6.5 (3.1–11.4)	5.2 (2.7–8.1)	7.6 (3.4–12.5)	<0.0001
**Male**, *n* (%)	274 (55)	93 (54.1)	181 (55.5)	0.7768
**Diagnosis**				
- ALL	409 (82.1)	140 (81.4)	269 (82.5)	0.8059
- AML	89 (17.9)	32 (18.6)	57 (17.5)	
**TCVC use for**				
- first therapy	399 (80.1)	156 (90.7)	243 (74.5)	< 0.0001
- first relapse	75 (15.1)	13 (7.6)	62 (19.0)	
- second relapse	12 (2.4)	–	12 (3.7)	
- third relapse	1 (0.2)	1 (0.6)	–	
- multiple disease stages	11 (2.2)	–	–	
**TCVC type**				
- Groshong	340 (68.3)	104 (60.5)	236 (72.4)	0.0038
- Hickman-Broviac	129 (25.9)	60 (34.9)	69 (21.2)	
- Port	29 (5.8)	8 (4.7)	21 (6.4)	
**Catheter diameter (French)**				
- ≤7	278 (55.8)	114 (66.3)	164 (50.3)	0.0006
- >7	220 (44.2)	58 (33.7)	162 (49.7)	
**Number of catheter lumens**				
- Single lumen	273 (54.8)	106 (61.6)	167 (51.2)	0.0266
- Double lumen	225 (45.2)	66 (38.4)	159 (48.8)	
**Catheter days (CD), Sum**	99,681	35,170	64,511	–

### Primary Outcome

**Figure 1:**
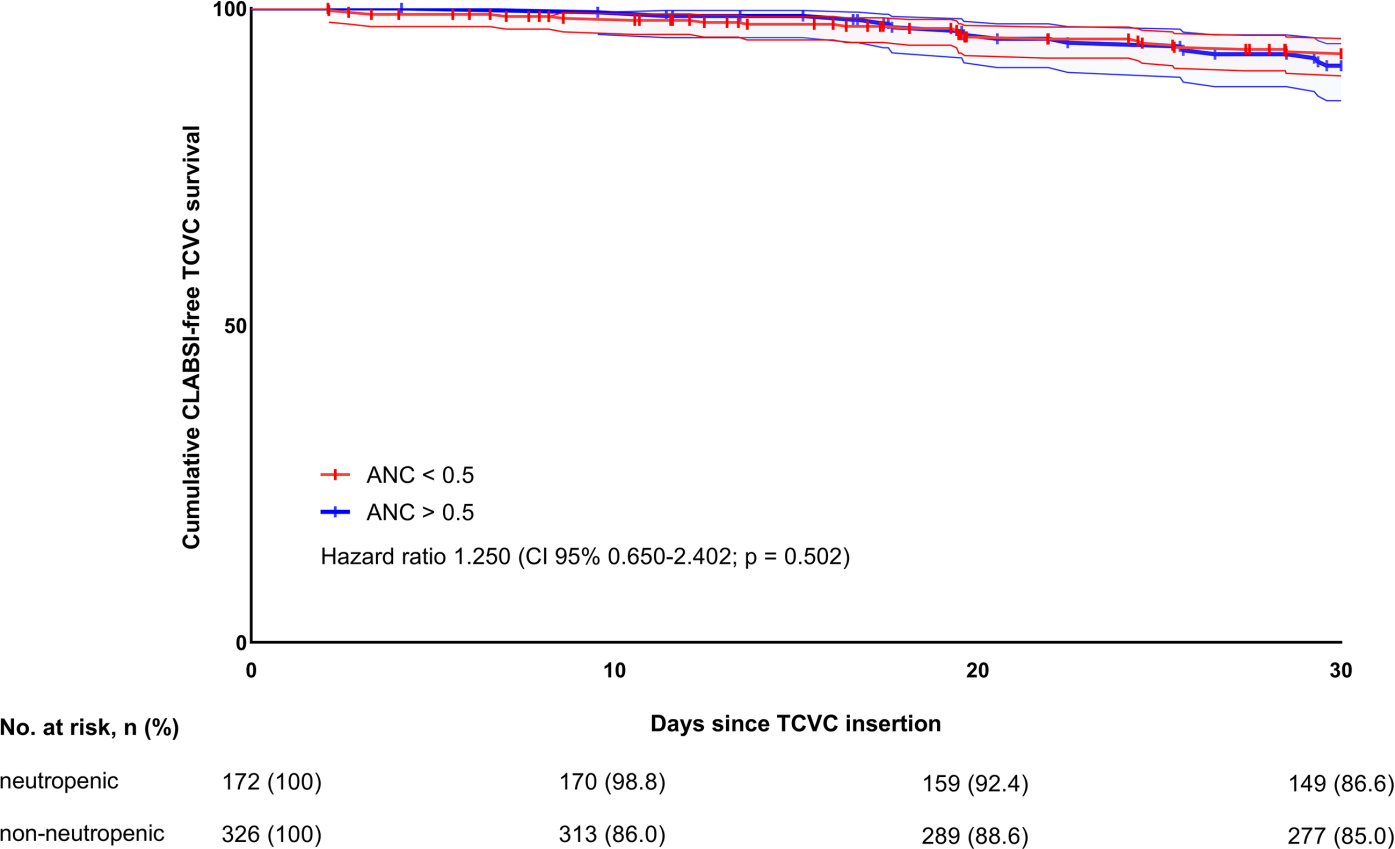
Thirty-day CLABSI-free TCVC survival in neutropenic and non-neutropenic patients.

In total, 165 CLABSI episodes (CR = 1.65 per 1,000 CD [99,681 days]) during the overall study period were observed, including 127 (76.9%) first CLABSI, 29 (17.6%) first recurrent, 7 (4.2%) second recurrent, and 2 (1.2%) third recurrent CLABSI episodes. In the neutropenic at implantation cohort, we found 47/165 (28.5%) CLABSI episodes, of which 35 (74.4%) were first CLABSI, 8 (17.0%) first recurrent, 3 (6.4%) second recurrent, and 1 (2.1%) third recurrent CLABSI. In non-neutropenic patients, there were 118/165 (69.2%) CLABSI episodes, of which 92 (77.9%) were first CLABSI, 21 (17.8%) first recurrent, 4 (3.4%) second recurrent, and 1 (0.8%) third recurrent. Within the first 30 days after TCVC placement, there were 37 (first) CLABSI episodes (15 [40.5%] CLABSI occurred in neutropenic and 22 [59.5%] in non-neutropenic at implantation patients, **Supplemental Figure S1**) and 4 CESI (all in non-neutropenic at implantation patients). All CESI culminated in premature catheter removal, whereas all of the CLABSI episodes were successfully managed conservatively with antibiotics. Perioperative neutropenia did not influence the development of early CLABSI in univariable (HR 1.250, 95% CI 0.650–2.402, *p* = 0.502) and multivariable competing-risks regression models (HR 1.633, 95% CI 0.846–3.152, *p* = 0.143) (**Figure 1**). Among the other investigated potential risk factors contributing to early CLABSI, female sex (HR 2.640, 95% CI 1.328–5.250, *p* = 0.006) and the use of TCVC for the treatment of relapsed leukemia (HR 4.347, 95% CI 2.283–8.264, *p* < 0.0001) were associated with higher CLABSI rates when using univariable models. This association was also confirmed in multivariable regression model (female sex, HR 2.444, 95% CI 1.227–4.865, *p* = 0.011; TCVC use for relapsed leukemia, HR 4.032, 95% CI 1.519–10.638, *p* = 0.005) indicating both variables as independent risk factors for early CLABSI (**Table 2**).

**Table 2:** Univariable and multivariable regression analysis for primary outcome.

	Early CLABSI (30 days)			
	Hazard ratio (95% CI), *p*-value			
	Univariable models		Multivariable models	
	Traditional	CFI	Traditional	CFI
**Age**	1.040 (0.975–1.109), 0.236	1.039 (0.973–1.110), 0.244	1.035 (0.955–1.121), 0.404	1.0355 (0.962–1.114), 0.352
**Sex** (female *vs.* male)	2.638 (1.326–5.263), 0.006	2.640 (1.328–5.250), 0.006	2.427 (1.199–4.926), 0.014	2.444 (1.227–4.865), 0.011
**Diagnosis** (AML *vs.* ALL)	1.519 (0.717–3.218), 0.276	1.518 (0.716–3.217), 0.276	1.336 (0.614–2.915), 0.464	1.343 (0.579–3.116), 0.492
**TCVC use** (therapy for relapse *vs.* first therapy)	4.339 (2.276–8.270), < 0.0001	4.347 (2.283–8.264), < 0.0001	4.045 (1.765–9.270), 0.001	4.032 (1.519–10.638), 0.005
**TCVC type** (Groshong, Hickman-Broviac, Port catheter)	1. Ref. 1.366 (0.607–2.942), 0.471 0.885 (0.210–3.721), 0.867	1. Ref. 1.307 (0.609–2.801), 0.492 1.063 (0.7508–2.221), 0.871	1. Ref. 0.552 (0.102–2.988), 0.491 0.642 (0.072–5.732), 0.691	1. Ref. 0.728 (0.358–1.481), 0.382 0.931 (0.448–1.936), 0.849
**Catheter diameter (French)** (≤7 *vs*. >7)	2.232 (1.148–4.329), 0.018	2.230 (1.150–4.323), 0.018	1.460 (0.284–7.498), 0.651	1.317 (0.366–4.739), 0.673
**Number of catheter lumens** (DL *vs.* SL)	2.681 (1.346–5.319), 0.005	2.677 (1.348–5.318), 0.005	1.838 (0.514–6.578), 0.349	1.720 (0.540–5.473), 0.358
**ANC at TCVC placement** (<0.5 *vs.* >0.5)	1.250 (0.648–2.409), 0.504	1.250 (0.650–2.402), 0.502	1.642 (0.830–3.246), 0.154	1.633 (0.846–3.152), 0.143

### Secondary Outcomes

#### CLABSI Incidence During Overall Study Period

**Figure 2:**
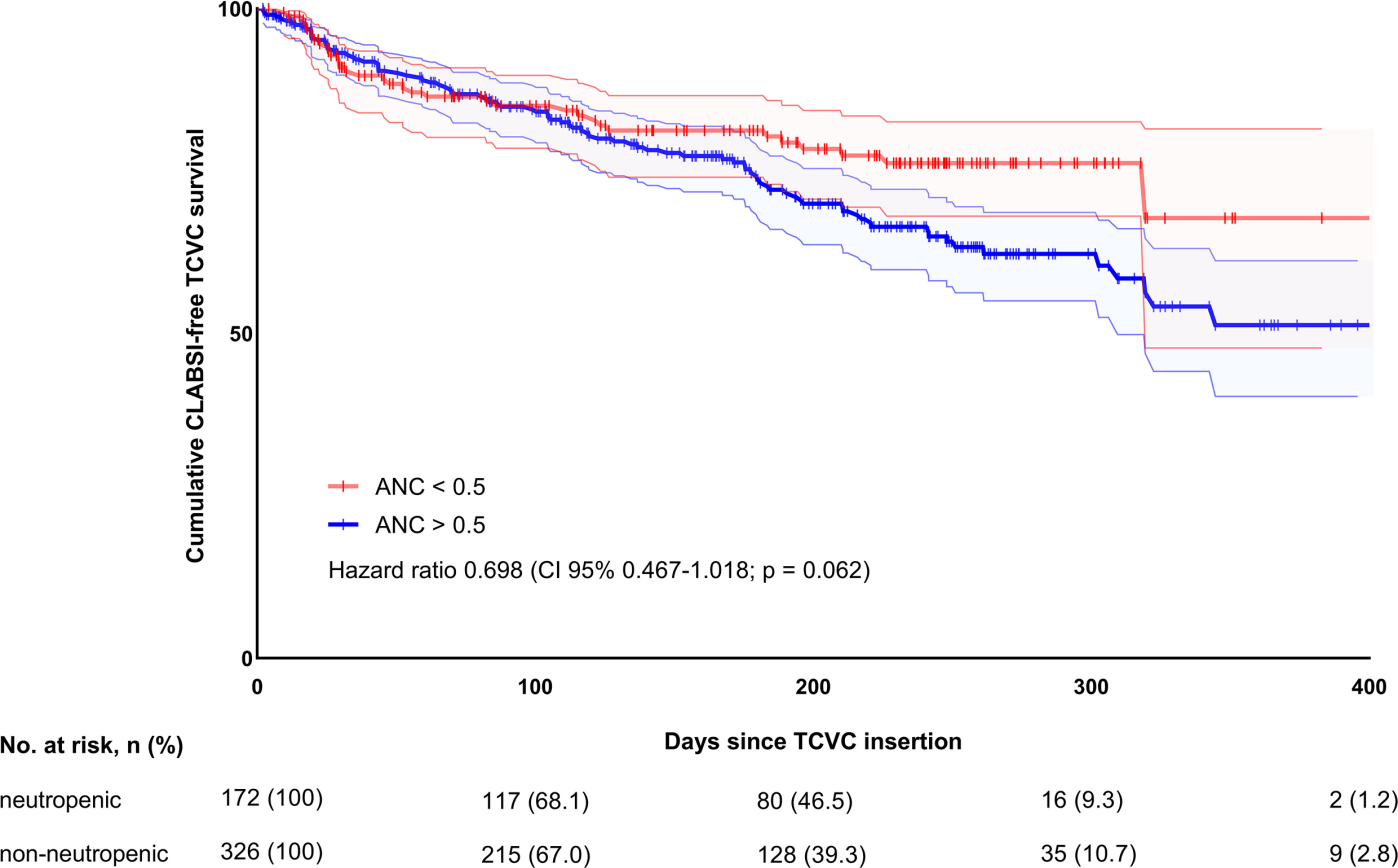
Overall CLABSI-free TCVC survival in neutropenic and non-neutropenic patients.

During the overall study period, the CLABSI-free TCVC survival was estimated at 76% within the neutropenic and 71% within the non-neutropenic at implantation cohort (**Supplemental Figure S2**). Among 127 first CLABSI events, 110 were successfully managed conservatively and 17 (13.4%) required premature catheter removal. Univariable and multivariable regression models revealed no differences in the (first) CLABSI rates between patients with and without perioperative neutropenia (HR 0.698, 95% CI 0.472–1.024, *p* = 0.066; HR 0.895, 95% CI 0.596–1.344, *p* = 0.594) (**Figure 2**). Among other potential risk factors, patients age at time of TCVC insertion (HR 1.053, 95% CI 1.016–1.091, *p* = 0.004), AML as underlying disease (HR 1.636, 95% CI 1.070–2.501, *p* = 0.023), TCVC use for treatment of relapsed leukemia (HR 3.215, 95% CI 2.207–4.694, *p* < 0.0001), the use of Groshong catheter (HR 1.626, 95% CI 1.055–2.500, *p* = 0.028), the use of double-lumen catheters (HR 3.335, 95% CI 2.316–4.801, *p* < 0.0001), and the use of small catheter (≤ 7 French, HR 2.825, 95% CI 1.973–4.041, *p* < 0.0001) were found to increase CLABSI rates in the univariable model. While the effect of TCVC use for treatment of relapsed leukemia (HR 2.004, 95% CI 1.240–3.326, *p* = 0.005) and the use of double-lumen catheters persisted in the multivariable regression model (HR 2.607, 95% CI 1.392–4.880, *p* = 0.003), the variables, which were found to be significant in univariable model, were no longer significant (**Table 3**).

**Table 3:** Univariable and multivariable regression analysis for secondary outcome.

	Overall CLABSI			
	Hazard ratio (95% CI), *p*-value			
	Univariable models		Multivariable models	
	Traditional	CFI	Traditional	CFI
**Age**	1.053 (1.017–1.091), 0.004	1.053 (1.016–1.091), 0.004	1.019 (0.977–1.064), 0.377	1.019 (0.976–1.064), 0.376
**Sex** (female *vs.* male)	1.094 (0.771–1.550), 0.615	1.093 (0.770–1.552), 0.617	1.039 (0.726–1.485), 0.833	0.727 (0.696–1.497), 0.816
**Diagnosis** (AML *vs.* ALL)	1.636 (1.072–2.493), 0.022	1.636 (1.070–2.501), 0.023	1.319 (0.845–2.057), 0.223	1.324 (0.845–2.074), 0.219
**TCVC use** (therapy for relapse *vs.* first therapy)	3.214 (2.221–4.651), < 0.0001	3.215 (2.207–4.694), < 0.0001	2.007 (1.289–3.123), 0.002	2.004 (1.240–3.326), 0.005
**TCVC** type (Groshong, Hickman-Broviac, Port catheter)	1. Ref. 1.638 (1.053–2.547), 0.029 1.280 (0.560–2.926), 0.558	1. Ref. 1.626 (1.055–2.500), 0.028 1.132 (0.732–1.751), 0.579	1. Ref. 0.698 (0.261–1.868), 0.474 0.760 (0.215–2.688), 0.670	1. Ref. 0.792 (0.414–1.516), 0.483 1.502 (0.904–2.497), 0.116
**Catheter diameter (French)** (≤7 *vs.* >7)	2.824 (1.964–4.065), <0.0001	2.825 (1.973–4.041), <0.0001	1.139 (0.452–2.865), 0.782	1.061 (0.535–2.109), 0.865
**Number of catheter lumens** (DL *vs.* SL)	3.333 (2.304–4.830), <0.0001	3.335 (2.316–4.801), <0.0001	2.739 (1.288–5.847), 0.009	2.607 (1.392–4.880), 0.003
**ANC at TCVC placement** (<0.5 *vs.* >0.5)	0.698 (0.467–1.018), 0.062	0.698 (0.472–1.024), 0.066	0.898 (0.595–1.355), 0.609	0.895 (0.596–1.344), 0.594

#### Organisms Isolated at First CLABSI in Neutropenic and Non-Neutropenic Patients

Overall, 28 different bacterial/fungal strains were isolated from 127 blood cultures including 140 isolates (**Supplemental Table S1**). There were 116 (91.3%) monomicrobial and 11 (8.7%) polymicrobial CLABSIs. The most commonly isolated bacteria were *Viridans streptococci* (*n* = 30, 21.4%) followed by *E. coli* (*n* = 16, 11.4%) and *coagulase negative staphylococci* (*n* = 14, 10%). There were no differences in distribution of bacteria/fungi between neutropenic and non-neutropenic patients (both at OP and at CLABSI) when analyzing CLABSIs occurring within the first 30 days after TCVC placement. When focusing on overall study period, there were significant differences in bacterial distribution among Gram-positive species between neutropenic and non-neutropenic at CLABSI patients (dominance of *V. streptococci* [*n* = 30, 27.0%] in the neutropenic group, *p* = 0.009).

## Discussion

In this observational study, we retrospectively evaluated the association between perioperative neutropenia and other potential risk factors on development of CLABSI among a large and homogeneous cohort of pediatric patients with ALL and AML undergoing placement of TCVC. We demonstrated that ANC < 0.5 × 10^9^/L at the time of catheter placement was not associated with increased CLABSI incidence in either short-termor the overall study follow-up periods. We also showed that in the overall cohort of neutropenic and non-neutropenic at implantation patients, female sex and TCVC use in children with relapsed leukemia contributed to the development of CLABSI during the first 30 days after TCVC insertion. Investigating risk factors contributing to CLABSI during the overall study period, the use of double-lumen catheters and TCVC during leukemia relapse was found to increase the hazard for CLABSI.

Our finding that periprocedural neutropenia did not influence the risk for CLABSI is consistent with numerous previous reports (10, 19–22). Conversely, other studies evaluating the impact of perioperative neutropenia on catheter-associated infections have yielded a higher rate of CLABSI episodes in neutropenic children, thus proposing exclusion of these patients from TCVC placement and insertion of an alternative central venous access device, such as peripherally inserted central venous catheter (PICC) or percutaneous CVCs as a bridging strategy (7, 8, 23). However, comparison of our study findings to previously published reports is problematic because of a large heterogeneity within included study populations (e.g., different hematooncological diseases, lymphoma, and solid tumors).

In our study, we found early CLABSI events in 7.4% (37/498) and overall CLABSI events in 33.1% (165/498) of TCVC. This is very similar to a previous study investigating the incidence rate of late CLABSIs in children with cancer, reporting an overall rate of 29% for infectious episodes (24).

The CR for CLABSI during the overall study period was 1.65 per 1,000 CD [99,681 days]. This is in line with previous studies reporting pooled CR rates for different hematological malignancies and solid tumors ranging between 1.6 and 3.1 per 1,000 CD (25–29) and even below the published rate of 2.3 of the American National Healthcare Safety Network for the years 2006–2008 (30). In particular, children with acute leukemias were shown to have a 3.7- to 14.9-fold increased risk for catheter infections compared to patients with solid malignancies (12, 25). Thus, in our study, we observed a remarkably low CLABSI rate in children with acute leukemias.

We additionally studied other factors potentially contributing to early CLABSI development and found that female sex (HR 2.640, 95% CI 1.328–5.250, *p* = 0.006) and the use of TCVC for therapy of relapsed leukemia (HR 4.347, 95% CI 2.283–8.264, *p* < 0.0001) increase the hazard for TCVC-associated infections. The unexpected finding that girls showed a higher risk for early CLABSI was not previously observed by other authors (31). The finding that children with relapsed malignancies face an elevated risk for CLABSI is consistent with the results of a prospective multicenter study involving pediatric cancer patients (32). This higher risk may be related to the treatment intensity in patients who are vulnerable to the side effects of antineoplastic therapy due to the accumulating effect of organ toxicities after receiving first-line therapy (33, 34).

We also found that the use of dual-lumen TCVCs was associated with an increased probability for CLABSI development during the overall study period (HR 2.607, 95% CI 1.392–4.880, *p* = 0.003). These findings are comparable with the HR ranging from 2.4 (95% CI, 1.6–3.5) to 3.13 (95% CI 2.11–4.65) for double-lumen catheter compared to single-lumen devices found in the literature (35, 36).

In contrast to several other studies reporting a higher incidence of TCVC-associated infections in younger patients (22, 37), in children with AML (38) or in patients with tunneled externalized catheters compared to ports (27, 39), we found no influence of patient´s age, underlying type of leukemia, or device type on early or overall CLABSI episodes in our study when analyzing using multivariable regression models. One possible explanation for the lack of age dependency on CLABSI risk observed in our study is that, in the regression models, we used age as continuous data and did not perform any categorization or dichotomizing of the patients. This would be arbitrary because the cutoffs are not defined, leading to loss of information or increasing bias effect (40, 41). Regarding the lack of association between type of underlying disease and CLABSI rates, it is important to emphasize that intensity and duration of anticancer therapy vary significantly between different biologically distinct subtypes of ALL and AML (42–44), thus potentially contributing to the variation of infectious complications within the subgroups.

In the present study, the predominant organisms detected in neutropenic at CLABSI patients were *Viridans group streptococci* (VGS), which represent a part of the normal flora of the gastrointestinal tract. In neutropenic conditions, these organisms are able to translocate through compromised mucosal barriers of the gastrointestinal tract into the bloodstream. Our results of VGS in the setting of neutropenia at CLABSI onset are consistent with earlier studies demonstrating that VGS are the most common isolates in neutropenic adult and pediatric oncological study populations (45, 46).

We are cognizant of the several limitations of this study. First, the retrospective nature and limitation to a single center involving institutional-specific treatment preferences and practices could have an effect on the generalizability of the study results. Second, although we used well-defined criteria of CLABSI (LCBI 1 criteria), the clinical characteristics of the patients (LCBI 2 criteria) were not considered, as no sign or symptom such as fever are needed to meet LCBI 1 criteria. Moreover, important clinical information such as severity of CLABSI is lacking. Nevertheless, we believe that in a setting of retrospective data collection, CLABSI definition according to LCBI 1 criteria is more suitable and robust. From an epidemiological point of view, because of the application of LCBI 1 criteria (eligible central line is a central line that has been in place for more than two consecutive calendar days), none of the study participants was in effect at risk for the study outcome during the first 48 h after TCVC placement (immortal person-time), which could result in an underestimated cumulative rate of the first CLABSI reported by the current study. Third, due to variations in frequency of blood sample collection during the further treatment course, we were not able to sufficiently track the ANC values from the time point of TCVC insertion until CLABSI onset. Consequently, it was not possible to adjust the CLABSI incidence to duration of neutropenia. However, we adjusted CLABSI incidence to 1,000 utilization days for TCVC. Finally, given that the purpose of this study was to determine the risk factors for CLABSI, we did not consider the treatment options for CLABSI. Notwithstanding, we believe that our study provides robust evidence on risk factors contributing to CLABSI in hematooncological patients with ALL and AML thanks to application of a strict CLABSI definition, inclusion of a large and homogeneous patient series, and conduction of a univariable and multivariable analysis minimizing interactions between clinical and device characteristics.

In conclusion, we showed that a perioperative ANC < 0.5 × 10^9^/L does not impact the incidence of early and overall CLABSI episodes in pediatric patients with acute leukemia. We also showed that the use of double-lumen catheters and the use of TCVC for treatment of relapsed leukemia were independent risk factors for CLABSI development. Efforts should be made to reduce CLABSI incidence through more intensive surveillance by implementing catheter care programs.

## Data Availability Statement

The raw data supporting the conclusions of this article will be made available by the authors, without undue reservation.

## Ethics Statement

The study protocol was approved by the Institutional Review Board of the University Hospital of Freiburg on December 8, 2020 (IRB, protocol number 20-1257). Written or verbal informed consent was not required. Written informed consent for participation was not provided by the participants’ legal guardians/next of kin because written or verbal informed consent was not required.

## Author Contributions

JS and IM conceptualized and designed the study. JS collected data. IM carried out the statistical analyses and drafted the initial manuscript. JS and IM reviewed and revised the manuscript. Both authors approved the final manuscript as submitted and agree to be accountable for all aspects of the work.

## Funding

The article processing charge was funded by the Baden-Wuerttemberg Ministry of Science, Research and Art and the University of Freiburg in the funding programme Open Access Publishing.

## Conflict of Interest

The authors declare that the research was conducted in the absence of any commercial or financial relationships that could be construed as a potential conflict of interest.

## Publisher’s Note

All claims expressed in this article are solely those of the authors and do not necessarily represent those of their affiliated organizations, or those of the publisher, the editors and the reviewers. Any product that may be evaluated in this article, or claim that may be made by its manufacturer, is not guaranteed or endorsed by the publisher.

## Supplementary Material

Supplementary Figure 1 CLABSI incidence during first 30 days after TCVC placement.

Supplementary Figure 2 CLABSI incidence during overall study period.

Supplementary Table 1 Microorganisms isolated at CLABSI onset stratified by ANC status at TCVC insertion/CLABSI onset and follow up period.
