# Supplementary figures and images for: Impact of Perioperative Absolute Neutrophil Count on Central Line-Associated Bloodstream Infection in Children With Acute Lymphoblastic and Myeloid Leukemia
Source: Front Oncol. 2021 Nov 23;11:770698. doi: 10.3389/fonc.2021.770698 (PMC8649799; doi:10.3389/fonc.2021.770698)

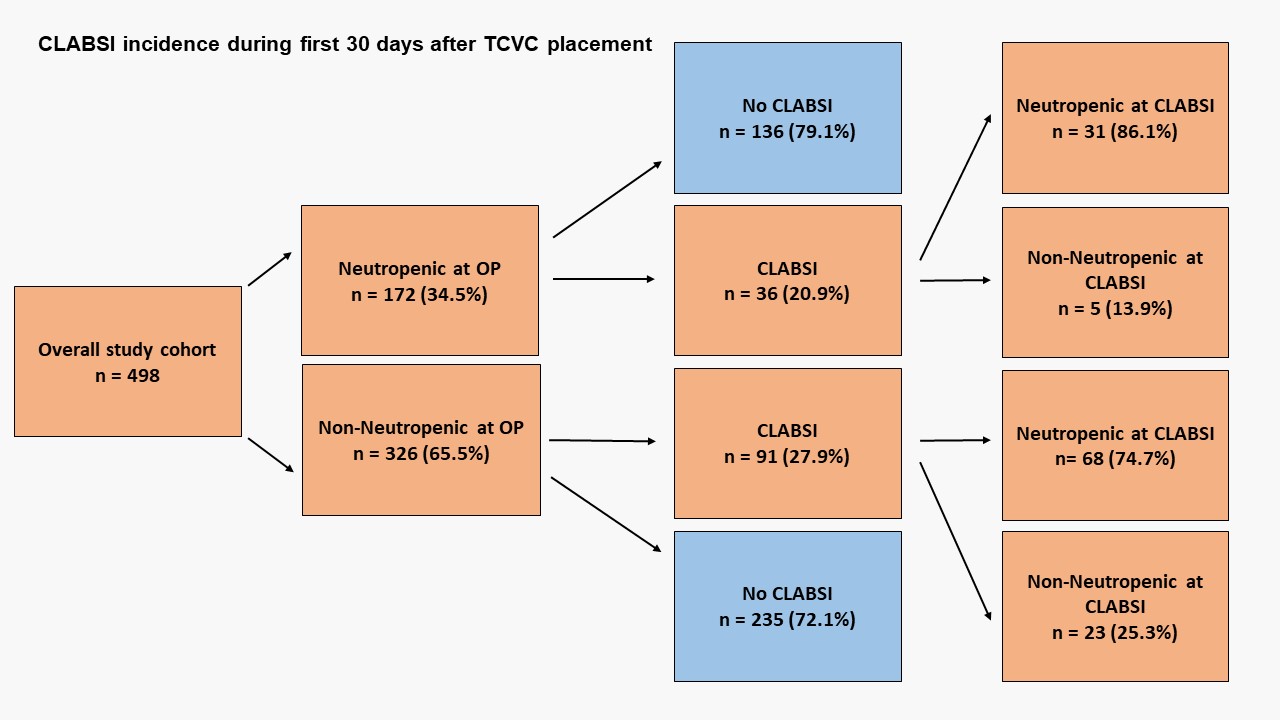

Supplement: Supplementary Figure 1 — CLABSI incidence during first 30 days after TCVC placement. [file Image_1.jpeg]

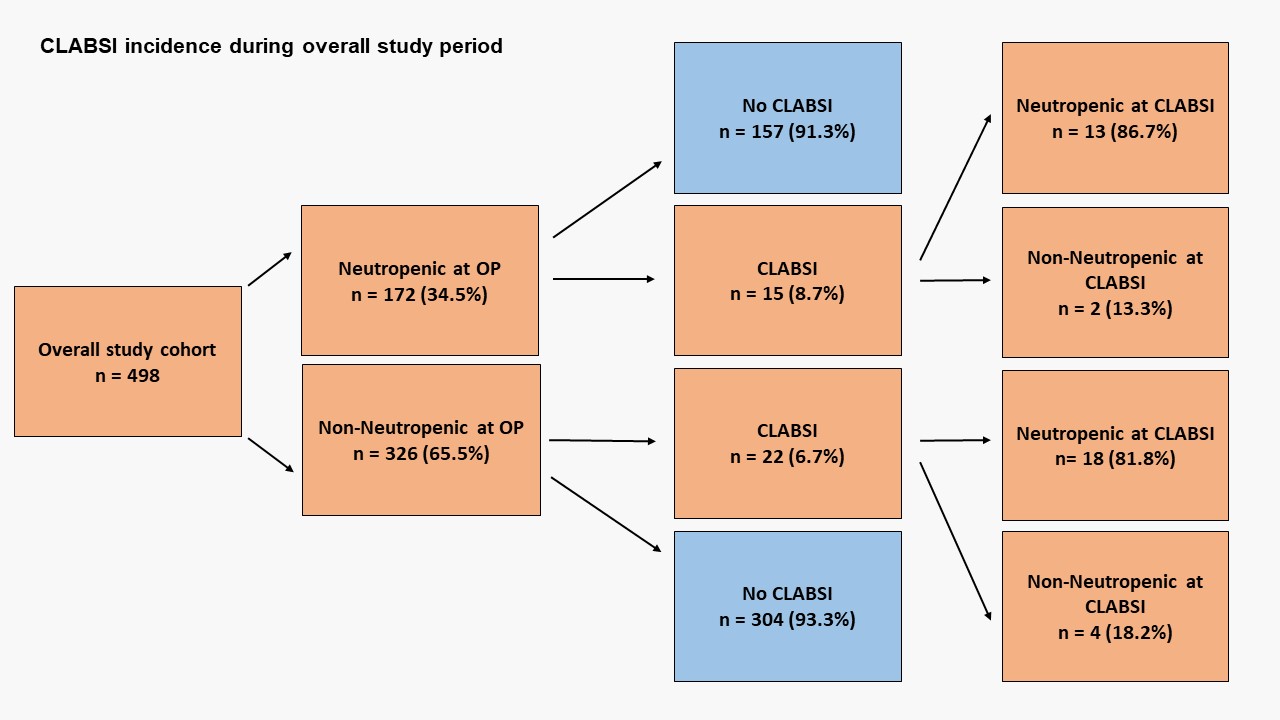

Supplement: Supplementary Figure 2 — CLABSI incidence during overall study period. [file Image_2.jpeg]
