# Supplementary material for: Impact of Perioperative Absolute Neutrophil Count on Central Line-Associated Bloodstream Infection in Children With Acute Lymphoblastic and Myeloid Leukemia
Source: Front Oncol. 2021 Nov 23;11:770698. doi: 10.3389/fonc.2021.770698 (PMC8649799; doi:10.3389/fonc.2021.770698)
Supplement: Supplementary Table 1 — Microorganisms isolated at CLABSI onset stratified by ANC status at TCVC insertion/CLABSI onset and follow up period. [file Table_1.docx]

**Supplemental TABLE 1 Microorganisms isolated at CLABSI onset stratified by ANC status at TCVC insertion/CLABSI onset and follow up period**

|  | **CLABSI onset within first 30 days after TCVC insertion**  **(n=37)** | | | | | | **CLABSI onset within overall study period**  **(n=127)** | | | | | |
| --- | --- | --- | --- | --- | --- | --- | --- | --- | --- | --- | --- | --- |
|  | **Neutropenic at TCVC insertion** | | **P value** | **Neutropenic at CLABSI onset** | | **P value** | **Neutropenic at TCVC insertion** | | **P value** | **Neutropenic at CLABSI onset** | | **P value** |
|  | **Yes**  **n = 15** | **No**  **n = 22** |  | **Yes**  **n = 31** | **No**  **n = 6** |  | **Yes,**  **n = 36** | **No,**  **n = 91** |  | **Yes**  **n = 99** | **No**  **n = 28** |  |
| **Isolates** | **n = 16** | **n = 25** | 0.453 | **n = 36** | **n = 6** | 0.929 | **n = 38** | **n = 102** | 0.557 | **n = 111** | **n = 29** | 0.019 |
| **Gram-positive bacteria** |  |  | 0.390 |  |  | 0.843 |  |  | 0.723 |  |  | 0.009 |
| *Enterococcus faecium* | 1 (6.2) | 1 (4.0) |  | 2 (5.5) | 0 (0.0) |  | 1 (2.6) | 3 (3.0) |  | 4 (3.6) | 0 (0.0) |  |
| *Enterococcus faecalis* | 0 (0.0) | 0 (0.0) |  | 0 (0.0) | 0 (0.0) |  | 0 (0.0) | 1 (1.1) |  | 1 (0.9) | 0 (0.0) |  |
| *CoNS* | 0 (0.0) | 4 (16.0) |  | 3 (9.7) | 1 (16.7) |  | 1 (2.6) | 13 (12.7) |  | 10 (9.1) | 4 (13.8) |  |
| *Staphylococcus aureus* | 0 (0.0) | 3 (12.0) |  | 2 (5.5) | 1 (16.7) |  | 1 (2.6) | 6 (5.9) |  | 5 (4.5) | 2 (6.9) |  |
| *Staphylococcus epidermidis* | 1 (6.2) | 0 (0.0) |  | 1 (3.2) | 0 (0.0) |  | 2 (5.3) | 1 (1.1) |  | 3 (2.7) | 0 (0.0) |  |
| *Staphylococcus hominis* | 1 (6.2) | 1 (4.0) |  | 1 (3.2) | 1 (16.7) |  | 1 (2.6) | 4 (3.9) |  | 4 (3.6) | 1 (3.4) |  |
| *Staphylococcus haemolyticus* | 0 (0.0) | 0 (0.0) |  | 0 (0.0) | 0 (0.0) |  | 0 (0.0) | 1 (1.1) |  | 1 (0.9) | 0 (0.0) |  |
| *Streptococcus pneumoniae* | 0 (0.0) | 2 (8.0) |  | 1 (3.2) | 1 (16.7) |  | 0 (0.0) | 2 (2.0) |  | 1 (0.9) | 1 (3.4) |  |
| *Aerococcaceae* | 1 (6.2) | 0 (0.0) |  | 1 (3.2) | 0 (0.0) |  | 3 (7.9) | 4 (3.9) |  | 4 (3.6) | 3 (10.3) |  |
| *Gemella spp.* | 0 (0.0) | 0 (0.0) |  | 0 (0.0) | 0 (0.0) |  | 2 (5.3) | 2 (2.0) |  | 3 (2.7) | 1 (3.4) |  |
| *Lactococcus spp.* | 0 (0.0) | 0 (0.0) |  | 0 (0.0) | 0 (0.0) |  | 1 (2.6) | 1 (1.1) |  | 2 (1.8) | 0 (0.0) |  |
| *Micrococcales* | 1 (6.2) | 1 (4.0) |  | 2 (5.5) | 0 (0.0) |  | 2 (5.3) | 5 (4.9) |  | 4 (3.6) | 3 (10.3) |  |
| *Viridans streptococci* | 2 (12.5) | 3 (12.0) |  | 4 (11.1) | 1 (16.7) |  | 9 (23.7) | 21 (20.6) |  | 30 (27.0) | 0 (0.0) |  |
| *Bacillaceae* | 1 (6.2) | 2 (8.0) |  | 3 (9.7) | 0 (0.0) |  | 3 (7.9) | 7 (6.9) |  | 3 (2.7) | 7 (24.1) |  |
| *Gordonia spp.* | 0 (0.0) | 0 (0.0) |  | 0 (0.0) | 0 (0.0) |  | 0 (0.0) | 1 (1.1) |  | 1 (0.9) | 0 (0.0) |  |
| *Clostridiaceae* | 0 (0.0) | 0 (0.0) |  | 0 (0.0) | 0 (0.0) |  | 0 (0.0) | 2 (2.0) |  | 2 (1.8) | 0 (0.0) |  |
| **Gram-negative bacteria** |  |  | 0.453 |  |  | 0.837 |  |  | 0.264 |  |  | 0.163 |
| *Proteus mirabilis* | 0 (0.0) | 0 (0.0) |  | 0 (0.0) | 0 (0.0) |  | 0 (0.0) | 1 (1.1) |  | 0 (0.0) | 1 (3.4) |  |
| *Enterobacter cloacae* | 0 (0.0) | 0 (0.0) |  | 0 (0.0) | 0 (0.0) |  | 0 (0.0) | 1 (1.1) |  | 0 (0.0) | 1 (3.4) |  |
| *Escherichia coli* | 2 (12.5) | 4 (16.0) |  | 5 (13.9) | 1 (16.7) |  | 3 (7.9) | 13 (12.7) |  | 12 (10.8) | 4 (13.8) |  |
| *Klebsielle* | 1 (6.2) | 1 (4.0) |  | 2 (5.5) | 0 (0.0) |  | 4 (10.5) | 4 (3.9) |  | 7 (6.3) | 1 (3.4) |  |
| *Pseudomonadales* | 3 (18.7) | 1 (4.0) |  | 4 (11.1) | 0 (0.0) |  | 3 (7.9) | 2 (2.0) |  | 5 (4.5) | 0 (0.0) |  |
| *Stenotrophomonas maltophila* | 0 (0.0) | 0 (0.0) |  | 0 (0.0) | 0 (0.0) |  | 0 (0.0) | 1 (1.1) |  | 1 (0.9) | 0 (0.0) |  |
| *Neisseria subflava* | 1 (6.2) | 0 (0.0) |  | 1 (3.2) | 0 (0.0) |  | 1 (2.6) | 0 (0.0) |  | 1 (0.9) | 0 (0.0) |  |
| *Fusobacterium nucleatum* | 0 (0.0) | 1 (4.0) |  | 1 (3.2) | 0 (0.0) |  | 0 (0.0) | 3 (3.0) |  | 3 (2.7) | 0 (0.0) |  |
| *Capnocytophaga sputigena* | 0 (0.0) | 0 (0.0) |  | 0 (0.0) | 0 (0.0) |  | 0 (0.0) | 1 (1.1) |  | 1 (0.9) | 0 (0.0) |  |
| **Fungi** |  |  | - |  |  | - |  |  | - |  |  | - |
| *Candida krusei* | 0 (0.0) | 0 (0.0) |  | 0 (0.0) | 0 (0.0) |  | 0 (0.0) | 1 (1.1) |  | 1 (0.9) | 0 (0.0) |  |
| **Ohters** |  |  | - |  |  | - |  |  | - |  |  | - |
| *Non-fermenters* | 1 (6.2) | 0 (0.0) |  | 1 (3.2) | 0 (0.0) |  | 1 (2.6) | 0 (0.0) |  | 1 (0.9) | 0 (0.0) |  |
| *Group G streptococci* | 0 (0.0) | 1 (4.0) |  | 1 (3.2) | 0 (0.0) |  | 0 (0.0) | 1 (1.1) |  | 1 (0.9) | 0 (0.0) |  |
| **Polymicrobial infection** | 1 (x2) (6.2) | 3 (x2) |  | 4 (x2) | 0 (0.0) |  | 2 (x2)  (5.3) | 8 (x2)  1 (x3) |  | 9 (x2)  (8.1)  1 (x3) (0.9) | 1 (x2)  (3.4) |  |

Micrococcales: Rothia mucilaginosa, Micrococcus luteus; Gemella spp: Gemella morbillorum, Gemella haemolysans; Klebsielle: Klebsiella oxytoca; Lactococcus spp.: Lactococcus lactis cremoris, Lactococcus lactis; Pseudomonadales: Moraxella osloensis, Pseudomonas aeruginosa; Aerococcaceae: Abiotrophia defectiva; Bacillaceae: Bacillus pumilus.
Data shown as: n (% of isolates)
